# Combined Petrosectomy in a 7-Year-Old Patient: Case-Based Video Illustration and Technical Considerations for Petrosal Approaches in the Pediatric Population

**DOI:** 10.1227/neuprac.0000000000000241

**Published:** 2026-06-01

**Authors:** Veronica de los Santos, Arianna Fava, Gabriel Heemann, Alexandre Gehanno, Adam Biard, Thibault Passeri, Kevin Beccaria, Sebastien Froelich

**Affiliations:** 1 Department of Neurosurgery, Lariboisière Hospital, GHU APHP Nord, Assistance Publique – Hôpitaux de Paris, Université Paris Cité, Paris, France;; 2 Department of Neurosurgery, Necker Hospital, Assistance Publique – Hôpitaux de Paris, Université Paris Cité, Paris, France

**Keywords:** Combined petrosectomy, Dermoid cyst, Inner ear development, Pediatric skull base surgery, Petrosal approaches, Temporal bone maturation

## Abstract

**BACKGROUND AND IMPORTANCE:**

Combined petrosectomy is a well-established technique in adult skull base surgery, offering wide exposure and enabling complete resection of complex lesions. In pediatric patients, its use remains uncommon because of the lower incidence of skull base tumors and age-dependent anatomic limitations, particularly in the developing temporal bone. We present a case of combined petrosectomy in a 7-year-old child and review the developmental anatomy relevant to its feasibility in children.

**CLINICAL PRESENTATION:**

A 7-year-old girl presented with progressive gait imbalance, dysarthria, and cranial nerve deficits. MRI revealed a large petroclival dermoid cyst causing brainstem compression. A mini-combined petrosectomy was performed, achieving gross total resection including the capsule with no complications. Postoperative recovery was uneventful.

**CONCLUSION:**

This case demonstrates that combined petrosectomy can be performed safely and effectively in selected pediatric patients once age-related anatomic conditions approach those seen in adults, as confirmed by preoperative imaging. These anatomic changes directly influence the feasibility and safety of combined petrosectomy in children.

**ABBREVIATION:**
APA: anterior petrous apex.

Combined petrosectomy is a key skull base approach. In adults, it enables single-stage gross total resection of complex lesions.^1-10^ In children, it is less commonly used because of the lower incidence of skull base lesions and age-related changes of the developing temporal bone that affect the feasibility of this approach.^8,11-13^

In early childhood, limited resectable bone restricts its utility. With growth, anatomic proportions approximate those of adults, allowing adult techniques,^14-16^ although the precise age threshold remains undefined.

We present the case of a 7-year-old patient with a large petroclival dermoid cyst operated through a mini-combined petrosectomy, providing technical insights and a review of age-related anatomic changes relevant to this approach.

## CLINICAL PRESENTATION

A 7-year-old girl presented with progressive gait decline, sialorrhea, nasal voice, and reduced handwriting speed. Neurological examination showed a Glasgow Coma Scale of 15, dysphonia, nasal resonance, palate asymmetry, and cerebellar signs including dysarthria, truncal ataxia, and limb dysmetria.

**FIGURE 1.**
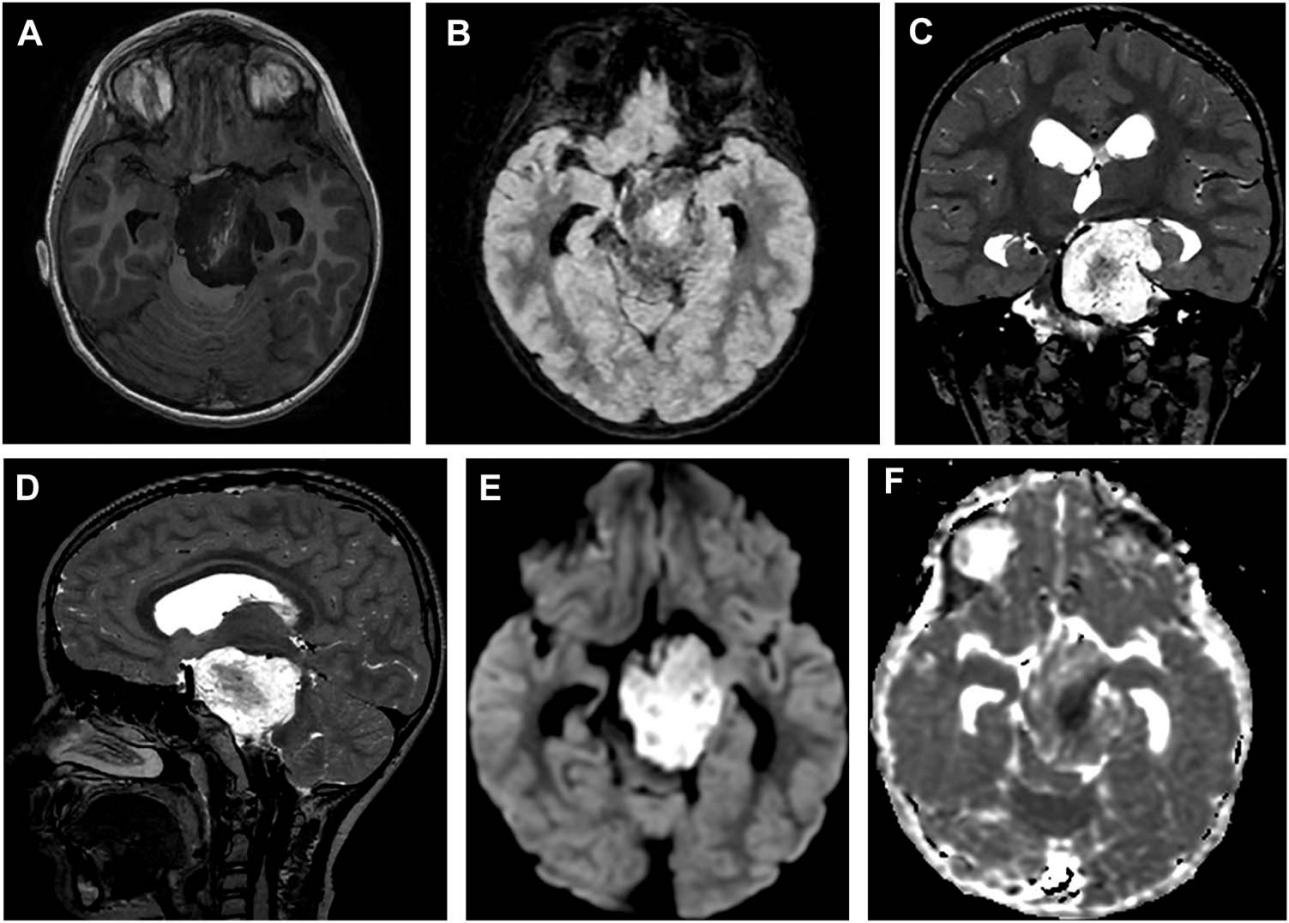
MRI demonstrates a well-defined extra-axial lesion in the petroclival region without contrast enhancement on axial T1-weighted imaging after gadolinium administration (**A**). The lesion exhibits incomplete suppression on axial fluid-attenuated inversion recovery (**B**) and displays a heterogeneous signal on coronal (**C**) and sagittal (**D**) T2-weighted imaging, characterized by central hypointense areas. Diffusion-weighted imaging (**E**) reveals marked restriction, with corresponding low signal on the apparent diffusion coefficient map (**F**). The overall heterogeneous appearance, with mixed T1 and T2 signal characteristics, is consistent with a dermoid cyst. This figure is original to this submission, so no credit or license is needed.

**FIGURE 2.**
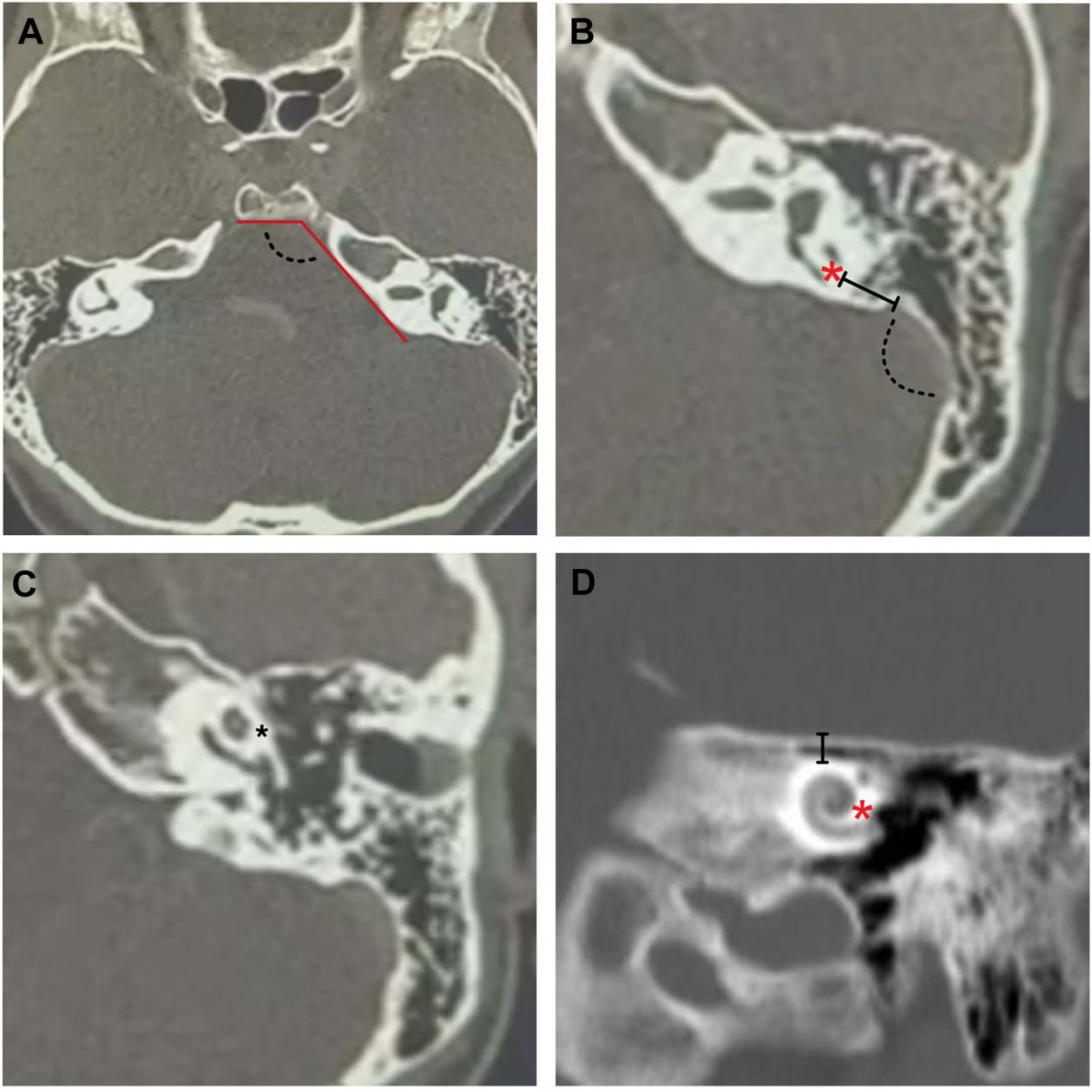
Preoperative high-resolution computed tomography scan demonstrating critical anatomic landmarks for surgical planning. **A**, Axial view illustrating the petroclival angle. **B**, Axial view showing the distance between the posterior semicircular canal and the posterior border of the petrous bone, with the dotted line indicating the topography of the lateral sinus. **C**, Axial view identifying the cochlea (black asterisk). **D**, Coronal view demonstrating the cochlea and the distance from the cochlea to the middle fossa floor. This figure is original to this submission, so no credit or license is needed.

Cerebral MRI (Figure 1) revealed a well-defined, extra-axial 5-cm lesion centered in the basal cisterns and petroclival region, with supratentorial and infratentorial extension, causing severe brainstem compression and obstructive hydrocephalus. The lesion was hypointense on T1-weighted imaging, heterogeneously hyperintense on T2-weighted imaging, and fluid-attenuated inversion recovery, without contrast enhancement, and showed diffusion-weighted imaging restriction, consistent with a dermoid cyst. Preoperative computed tomography scans (Figure 2) identified key structures, including the cochlea and semicircular canals, delineating the boundaries of a safe petrosectomy.

The patient was positioned supine with the head turned 70°. Cranial nerves and potentials were monitored. Neuronavigation identified the transverse-sigmoid junction. A question-mark incision was made, and a mini-combined petrosectomy was performed (Video) following the previously described technique,^4-10^ allowing safe tumor access, multiple lines of sight, a reduced surgical footprint, and single-stage gross total resection including the cyst wall, with endoscopic assistance for final visualization. No complications occurred.

**FIGURE 3.**
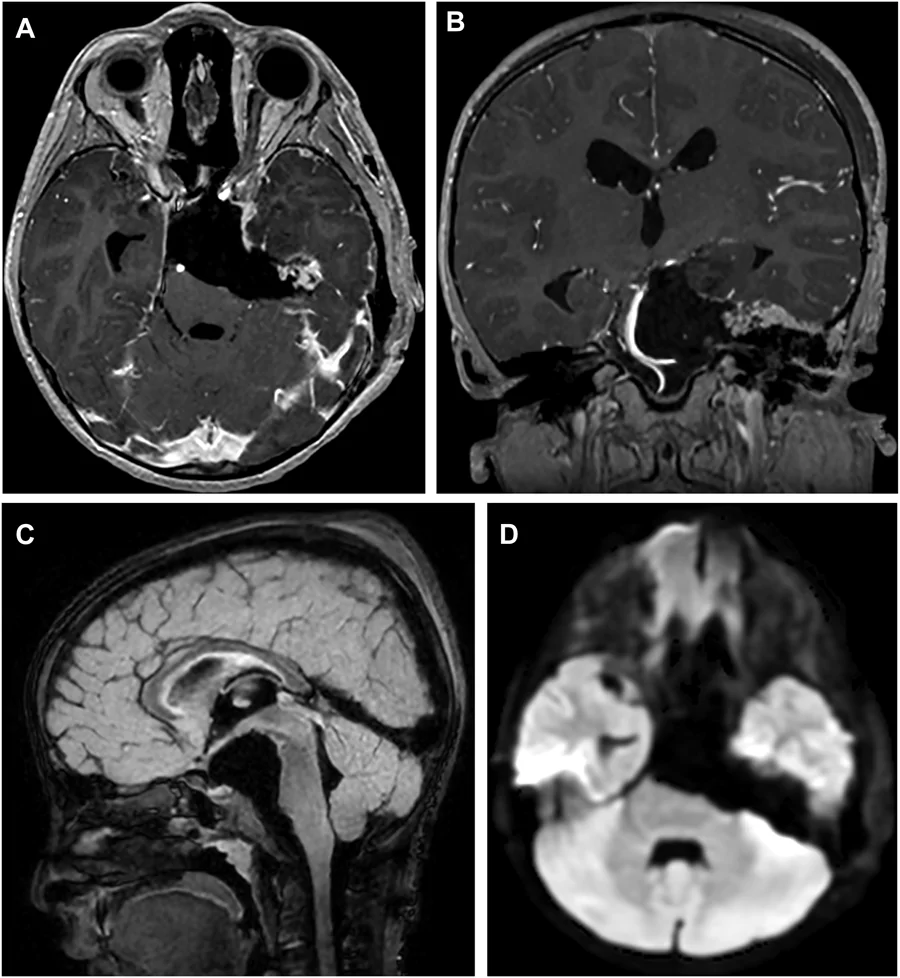
Postoperative MRI demonstrates complete resection of the dermoid cyst. Axial (**A**) and coronal (**B**) T1-weighted images and sagittal fluid-attenuated inversion recovery (**C**) show resolution of brainstem compression without residual lesion. Diffusion-weighted imaging (**D**) reveals no restricted diffusion, consistent with the complete removal of dermoid content. This figure is original to this submission, so no credit or license is needed.

Postoperative MRI confirmed gross-total resection (Figure 3). The postoperative course was uncomplicated. At discharge, the patient had a mild, incomplete left trochlear nerve palsy, which resolved within the first postoperative month. At 10-month follow-up, the patient was asymptomatic. A small 1-cm capsular remnant firmly adherent to the contralateral posterior communicating artery and the third cranial nerve is being closely monitored with serial imaging.

Institutional review board approval was not required by the institution. Parental consent for the procedure and for publication of the patient's data and images in the surgical video was obtained.

## DISCUSSION

This case demonstrates that combined petrosectomy can be safely used in selected pediatric patients with large or giant epidermoid or dermoid cysts of the petroclival region, aiming for complete cyst wall resection and potential cure at first surgery, which is critical for long-term control in this pathology.^17-19^ Residual cyst wall may lead to recurrence and reoperation, particularly in children, where repeated surgeries increase surgical complexity due to adhesions.

The above considerations support selecting a surgical approach that provides wide infratentorial and supratentorial exposure, allowing manipulation of critical neurovascular structures through multiple lines of sight. The Mini-combined transpetrosal approach offers an ascending trajectory that passes behind the labyrinth and below the temporal lobe, facilitating access to the upper petroclival and retroinfundibular regions without temporal lobe retraction. In addition, a descending trajectory through the petrous apex provides control of the lower prepontine and premedullary spaces, ipsilateral and contralateral.^5^

To our knowledge, this is the first published case of complete combined petrosectomy in a pediatric patient of this age. Few reports of anterior or posterior petrosectomy are available.^11-13,20-22^

From a certain age onward, anatomic conditions progressively approximate those encountered in adults. In our patient, imaging demonstrated sufficient petrous bone growth to allow expansion of surgical corridors around fixed inner ear structures, making combined petrosectomy a reasonable and safe option.

While no studies have specifically defined the age threshold at which this approach becomes truly advantageous, anatomic research on temporal bone development offers useful approximations. The petrous temporal bone develops through endochondral ossification and reaches near-adult dimensions around age 8 years.^23-26^ Several developmental changes influence the feasibility of pediatric petrosal approaches. The most relevant are the labyrinth–petrous bone relationship and the growth and pneumatization of the mastoid process and petrous apex.

### Labyrinth–Petrous Bone Relationship

Prenatal development of fully formed sensory organs is a key evolutionary adaptation that enhances postnatal survival by enabling immediate environmental interaction. Both the membranous and osseous labyrinth, along with the ossicles, reach adult dimensions by approximately the 20th week of gestation.^25,27-29^ Although inner ear structures are adult-sized at birth, the surrounding bone continues to grow postnatally.

**FIGURE 4.**
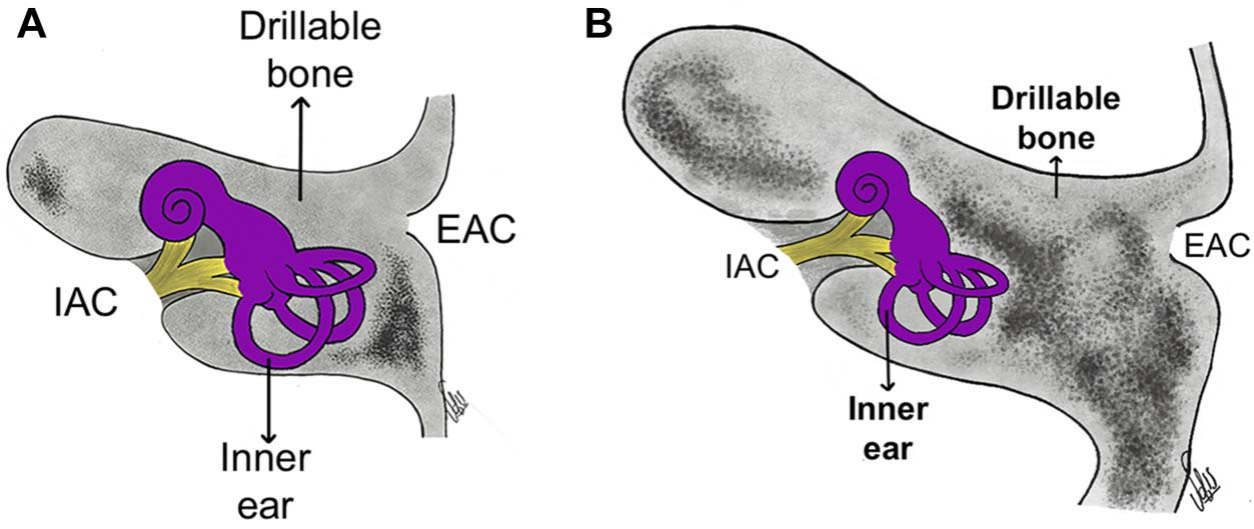
Evolution of labyrinth–petrous bone relationship in early childhood. **A**, Anatomic illustration at true scale of a newborn, showing the adult-sized inner ear (in purple) surrounded by a relatively small petrous bone. **B**, Representation of the same structures in a 7-year-old patient, illustrating the postnatal expansion of the petrous portion of the temporal bone. While inner ear structures remain constant in size, the growth of surrounding bone progressively increases the drillable space available for a petrosectomy. This age-dependent anatomical relationship is critical for planning safe surgical corridors. This figure is original to this submission, so no credit or license is needed. ICA, internal carotid artery; EAC, external auditory canal.

In petrosal approaches, the key anatomic variable is the ratio between the fixed inner ear structures and the available petromastoid bone volume for safe removal. As the child grows, this ratio becomes more favorable (Figure 4). Several methods evaluate this ratio. One compares the constant diameter of the basal cochlear turn with petrous bone length,^14^ evolving from 1:4 in infancy to 1:6 or more by adolescence. Another measures the distance from the posterior petrosal surface to the posterior semicircular canal, which increases with age and reflects drillable corridor expansion.^16^ This distance is also variable in adults.^30^

Growth patterns vary across the petrous bone: The posterior portion follows a more gradual trajectory, reaching adult dimensions between 12 and 15 years, while the anterior petrous apex (APA) matures earlier, completing its development between 8 and 11 years.^15,16^

In general, the literature suggests that key surgical landmarks used in adults for petrosectomy become reliably identifiable in children after 7 to 8 years.^13-16,23-25^

### Formation and Pneumatization of the Mastoid Process

Although pneumatization begins prenatally, the mastoid remains small and poorly defined in early life. Its growth accelerates during the first decade and continues into early adulthood.^25,31^ Limited mastoid pneumatization complicates the identification of landmarks such as the facial nerve and bony labyrinth. In young children, the facial nerve lies more superficially and inferiorly than in adults, increasing vulnerability. Incomplete mastoid development narrows the corridor between the sigmoid sinus and the labyrinth, limiting access.^26,30^

### Growth and Pneumatization of the Petrous Apex

The APA reaches adult dimensions between 8 and 11 years.^16,32^ In younger patients, the smaller APA volume may limit access to the most inferior petroclival region because of a more acute angle when drilling toward the lower posterior fossa.^16^

APA growth also affects the petroclival angle.^33^ In younger patients, this angle is typically more obtuse and progressively becomes more acute with age as the APA develops.^16,24^ This age-related variation should be considered when planning the approach and adapting patient positioning to optimize exposure.

### Other Considerations

The long-term effects of drilling on immature bone and hearing remain unclear. Concerns include disruption of ossification centers and sutures, potentially affecting growth, as well as the effects of high-intensity noise generated by drilling near the cochlea.^34^ Further studies are needed to assess these risks.

## CONCLUSION

Combined petrosectomy can be safely performed in selected pediatric patients with large or giant skull base epidermoid or dermoid cysts to achieve a cure. Age-dependent anatomic changes are critical, particularly the increasing ratio of drillable petrous bone to fixed inner ear structures, with direct surgical implications. Careful imaging is mandatory to confirm adequate anatomic maturity for safe drilling.

## Notes

**VIDEO.** Left mini-combined petrosectomy for resection of a petroclival dermoid cyst in a 7-year-old patient. The patient was positioned supine with the head rotated 70° and the vertex slightly down. A C-shaped incision around the ear was performed. Key technical steps included interfascial dissection before elevation of the temporalis muscle; neuronavigation-guided identification of the transverse-sigmoid sinus junction (TSSJ); C-shaped craniotomy with cosmetic mastoidectomy; posterior petrosectomy exposing the presigmoid dura, with identification of the superior semicircular canal (SSC), posterior semicircular canal (PSC), and peeling of the endolymphatic sac (ES); middle fossa peeling to expose the petrous apex; anterior petrosectomy; dural opening with ligation and section of the superior petrosal sinus; tentorial incision and opening of Meckel's cave; gross total resection of the cyst including its capsule with identification and preservation of neurovascular structures. Endoscopic assistance was used for final inspection before closure. Additional abbreviations (video): CSF, cerebrospinal fluid; EAC, external auditory canal; GSPN, greater superficial petrosal nerve; III CN, oculomotor nerve; III CN cont, contralateral oculomotor nerve; IIIrd V, third ventricle; IV CN, trochlear nerve; MMA, middle meningeal artery; ON, optic nerve; OQ, optic chiasm; SS, sigmoid sinus; TS, transverse sinus; V CN, trigeminal nerve; V3, mandibular nerve; VI CN, abducens nerve; ZP, zygomatic process.
